# The Role of Indigenous Yeasts in Shaping the Chemical and Sensory Profiles of Wine: Effects of Different Strains and Varieties

**DOI:** 10.3390/molecules29174279

**Published:** 2024-09-09

**Authors:** Xin-Ke Zhang, Pei-Tong Liu, Xiao-Wei Zheng, Ze-Fu Li, Jian-Ping Sun, Jia-Shuo Fan, Dong-Qing Ye, De-Mei Li, Hai-Qi Wang, Qing-Quan Yu, Zi-Yuan Ding

**Affiliations:** 1 Food Science and Engineering College, Beijing University of Agriculture, Beijing 102206, China; zhangxinke@bua.edu.cn (X.-K.Z.); demeili@sina.com (D.-M.L.); 2 “The Belt and Road” International Institute of Grape and Wine Industry Innovation, Beijing University of Agriculture, Beijing 102206, China; 3 Nutrition & Health Research Institute, COFCO Corporation, Beijing 102209, China; liupeitong@cofco.com (P.-T.L.); zhengxiaowei@cofco.com (X.-W.Z.); fanjiashuo@cofco.com (J.-S.F.); 4 COFCO Greatwall Chateau Sungod (Huailai) Co., Ltd., Zhangjiakou 075499, China; lzf@cofco.com (Z.-F.L.); sunjp@cofco.com (J.-P.S.); wanghaiqi1@cofco.com (H.-Q.W.); 5 Guangxi Key Laboratory of Fruits and Vegetables Storage-Processing Technology, Guangxi Academy of Agricultural Sciences, Nanning 530007, China; yedongqing@gxaas.net

**Keywords:** aroma compounds, GC-MS, sensory evaluation, rate-all-that-apply, multiple factor analysis

## Abstract

The microbial terroir is an indispensable part of the terroir panorama, and can improve wine quality with special characteristics. In this study, eight autochthonous yeasts (*Saccharomyces cerevisiae*), selected in Huailai country, China, were trailed in small-scale and pilot fermentations for both white (Riesling and Sémillon) and red (Cabernet Sauvignon and Syrah) wines and evaluated by GC-MS analysis and the rate-all-that-apply (RATA) method. Compared to commercial yeast strains, the indigenous yeasts were able to produce higher concentrations of ethyl esters and fatty acid ethyl esters, and higher alcohol, resulting in higher odor activity values of fruity, floral attributes. Marked varietal effects were observed in the pilot fermentation, but yeast strains exerted a noticeable impact in modulating wine aroma and sensory profile. Overall, indigenous yeast could produce more preferred aroma compounds and sensory characteristics for both white and red wines, demonstrating the potential for improving wine quality and regional characteristics.

## 1. Introduction

Innovative and premium wines are increasingly demanded by consumers worldwide. The production of a typical and characteristic wine in relation to a specific wine region challenges traditional enological ideology and technology. Currently, “terroir” is a well-accepted term first applied to viticulture, where it refers to the environmental conditions in which the grapes are grown, including the specific soil, topography, climate, landscape features, and human practices [1]. In fact, the biodiversity characteristics of a wine are also an indispensable part, and the “microbial terroir" is becoming a well-established concept used as a principal element in modern winemaking [2,3]. Microbial terroir is a collective concept that includes a wide range of microbial communities during viticulture and vinification, such as autochthonous yeasts (*Saccharomyces cerevisiae* or non-*Saccharomyces*), lactic acid, and even acetic acid bacteria [2]. However, these indigenous microbes have typically been replaced by commercial starter cultures in commercial winemaking, where a manageable, predictable, and reproducible product is desired. As a result, a continuous sensory profile can be observed in such wines despite their variety, origin, and viticultural practice [2], and the characteristics directly related to microbial terroir are diminished in such wines [4].

Recently, non-*Saccharomyces* has been a popular choice in research or winemaking practices that could improve the quality and complexity of wine. Although previously considered a spoilage microorganism, the role of non-*Saccharomyces* is now being re-evaluated and used as a biotechnological tool to compensate for traits absent in *S. cerevisiae* [2], such as reducing ethanol content [5], modulating wine acidity [6], and increasing color stability [7]. Despite these different purposes, the enhancement of wine flavor and aroma complexity is one of the main roles of non-*Saccharomyces* as they have different metabolomic systems compared to *S. cerevisiae*, resulting in high enzymatic activities related to the release of volatiles during fermentation [8,9]. However, with various oenological advantages, non-*Saccharomyces* generally run the risk of not being able to thoroughly complete alcoholic fermentation.

*S. cerevisiae* is generally capable of complete alcoholic fermentation and is therefore widely exploited and is even commercially developed [10,11,12]. Autochthonous *S. cerevisiae* showed better performance in modulating the sensory profile of red varieties of Vranec and Cabernet Sauvignon wine [13]. Similarly, autochthonous *S. cerevisiae* isolated from the Turkish native white cultivar Narince (*Vitis vinifera* cv.) exerted higher concentrations of acetates and ethyl esters, and achieved a better evaluation in floral and fruity attributes and the overall impression of the final wine [14]. Excellent organoleptic characteristics of Greek white wine of the Debina variety were obtained using indigenous *S. cerevisiae* compared to commercial yeast, and this was associated with the high expression of flavor-biosynthetic genes [15].

With the rapid growth in the wine market and industry, China is becoming a non-negligible participant in the wine world. In order to highlight the regional or sub-regional characteristics of wine, microbial diversities have been investigated and various indigenous yeast flora have been isolated, identified, and exploited from different wine regions [16], such as the northwestern wine regions of Ningxia Hui Autonomous Region [17] and the Xinjiang Uygur Autonomous Region [18,19], in the middle wine regions of Shaanxi province [20], in the eastern wine regions of Changli Country in Hebei province [21], and in Yantai city in Shandong province [22].

Huailai is one of the most famous wine regions in China, with over 1000 years of history of grape growing, and it is also the cradle of the modern domestic Chinese wine industry. Located in a mountainous area of Hebei province, only 120 km west-northwest of Beijing, Huailai has a continental semi-arid climate with abundant sunshine and rainfall, and is recognized as being able to produce the finest wine in China. However, the microbial biodiversity in Huailai has been barely investigated, and commercial yeast has been mostly prioritized, resulting in the wine of Huailai country having fewer regional characteristics. Recently, two autochthonous *S. cerevisiae* and non-*Saccharomyces* strains isolated in Huailai country showed good varietal aroma diversity of Marselan wine in a small-scale experiment [23], indicating the great microbial potential of this region. However, a comprehensive selection of indigenous yeasts in Huailai country or, to a greater extent, in China, is lacking. Therefore, this work carried out a systematic procedure of selecting a well-performing indigenous yeast in Huailai country, aiming to produce premium wine with regional characteristics. This work was achieved through a consecutive procedure of small-scale and pilot fermentation using various major white and red varieties in Huailai, together with comprehensive chemical analysis and sensory evaluation.

## 2. Results and Discussion

### 2.1. Fermentation Ability and Influences on the Main Chemical Composition of Wine

The fermentation ability of eight indigenous strains as well as a commercial yeast was evaluated in small-scale fermentation trails where the CO_2_ loss and OD_600_ value were measured (Table 1). ANOVA showed that there was no significant difference (*p* > 0.05) in CO_2_ loss and ethanol production for the nine yeast strains at the end of fermentation (Table 1), indicating that the eight selected yeasts had similar fermentation capability compared to the commercial wine yeast. The OD_600_ value of each strain increased throughout fermentation (Figure S1A in Supplementary Materials), indicating that the selected yeast strains were as active as the commercial yeast in a wine fermentation environment. Although the OD600 value of each strain varied significantly at the end of fermentation (Table 1), the fermentation capacity of all strains was similar, as similar CO_2_ loss curves were observed (Figure S1B in Supplementary Materials).

The major chemical composition of the wine, including glucose, fructose, glycol, and organic acids (including tartaric acid, malic acid, citric acid, succinic acid, lactic acid, and acetic acids) was also analyzed (Table 1). No glucose and low levels of fructose were detected for all strains (Table 1), confirming their good fermentative capacity. It is noteworthy that the concentrations of grape-derived organic acids, namely tartaric acid, malic acid, and citric acid, were at a similar level (Table 1). In general, it seems that these compounds were not easily influenced by the strains, except for significantly higher concentrations of tartaric acid and malic acid for strain 60,682, and significantly lower (*p* < 0.05) citric acid for the 60,673 and 60,685 strains (Table 1). Conversely, other compounds that were extracellular metabolites produced by the yeast during fermentation, namely, glycerol, succinic acid, lactic acid, and acetic acid, were significantly different (*p* < 0.05) between the selected strains and the commercial strain (Table 1). These compounds could modify the palate of wines. Nevertheless, the concentrations of these compounds were all within the normal range and would not have any adverse effect on wine quality [24].

### 2.2. Aroma Compound Production of Each Strain in Small-Scale Fermentation

**Figure 1:**
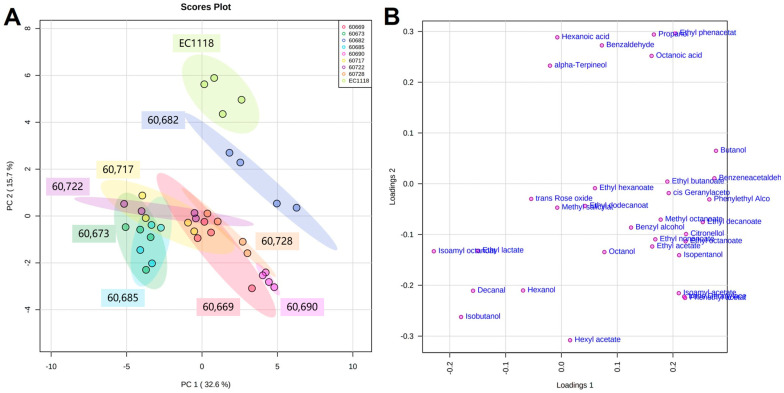
Principal component analysis (PCA) of the concentration of aroma compounds using indigenous and commercial yeasts in small-scale fermentation. (Indigenous strains: 60,669, 60,673, 60,717, 60,722, 60,728, 60,682, 60,685, 60,690; commercial strains: EC1118). (**A**), score plot of PCA; (**B**), loading plot of PCA.

To better evaluate the effects of yeast on the production of aroma compounds in the final wine, small-scale fermentation of each strain was performed using sterilized Sémillon must. A total of 33 aroma compounds were quantified, including higher alcohols, acetate esters, ethyl esters, medium-chain fatty acids (MCFAs), aldehydes, and terpenes (Table S1 in Supplementary Materials). Based on the concentration of these aroma compounds, PCA was used to interpret the characteristics of the yeast strains (Figure 1). The score plot was made using PC1 and PC2, which explained 48.3% of the total variance. The commercial yeast (EC1118) is clearly distinguished from the other indigenous yeast strains in the score plot in Figure 1A. Their separation was primarily on the PC2 axis, which was mainly attributed to ethyl phenylacetate, propanol, hexanoic acid, benzaldehyde, octanoic acid, and α-terpineol (Figure 1B). The concentration of these compounds was pronouncedly higher (not necessarily significantly) in the commercial yeast than in the indigenous yeast strains (Table S1 in Supplementary Materials). Among these compounds, octanoic acid and hexanoic acid belonging to MCFAs was above the threshold value (Table S1 in Supplementary Materials), indicating fewer fatty notes in the selected indigenous strains. The MCFAs were derived from the progressive 2-carbon elongation steps of fatty acid biosynthesis, which was strongly related to the synthesis of the precursor acyl-CoA and the expression of related genes, which was dominated by the yeast [25,26]. Despite the lower concentration of octanoic acid and hexanoic acid, their ethyl esters, namely, ethyl octanoate and ethyl hexanoate with a fruity note, did not show a notably lower concentration in the selected yeast strains (Table S1 in Supplementary Materials). This was possibly due to the different gene expressions of the yeast strains, especially for the expression of Eht1p and Eeb1p encoded by the gene EHT1, which was the key factor in modulating the final ethyl ester production [27].

The selected yeast strains showed similarities and distinctive characteristics in terms of aroma profile (Figure 1A). Strain 60,682 is located between the commercial yeast strain and the other indigenous yeast strains in Figure 1A, indicating that it had a similar aroma production ability as these two strains. In addition, although circled separately, the proximity of some indigenous yeasts in Figure 1A might indicate their similarity in terms of aroma profile. For example, stains 60,669, 60,728, 60,690 were close to each other, and strain 60,673 was adjacent to 60,685 (Figure 1A). The differentiation of these two groups was laid along the PC1 axis, where the most positively correlated variables were butanol, benzeneacetaldehyde, phenylethyl alcohol, ethyl decanoate, citronellol, ethyl octanoate, phenethyl acetate, trans-geranylacetone, isopentanol, isoamyl acetate, and ethyl phenylacetate, and the most negatively related variables were isoamyl octanoate, isobutanol, decanal, ethyl lactate, and hexanol (Figure 1B). This difference was possibly due to metabolomic differences of the yeast strains. Among these compounds, benzeneacetaldehyde, phenylethyl alcohol, isopentanol, and isobutanol were successively produced by the Ehrlich pathway using L-phenylalanine, and isoleucine and leucine as substrates, respectively [28,29]. In addition, the other maker, namely, butanol with a high positive loading in PC1, was synthesized using 2-keto acid precursors that were also dominated by the yeast metabolism [30]. The other esters, mostly classes of acetate esters of higher alcohols and ethyl esters of fatty acids, were closely related to the concentration of their precursor, such as higher alcohols and fatty acids, both of which were closely related to the yeast metabolism [25]. Among these compounds, benzeneacetaldehyde, phenylethyl alcohol, isopentanol, isoamyl acetate, and phenethyl acetate, which mainly contributed to floral, honey, sweet and tropic fruit characters [31], were remarkably higher in strains 60,669, 60,728 and 60,690 (Table S1 in Supplementary Materials). However, isobutanol, isoamyl octanoate, and ethyl lactate, which had chemical, fruity, and buttery characters [31], were more pronounced in strains 60,673 and 60,685 (Table S1 in Supplementary Materials).

In Figure 1A, strains 60,717 and 60,722 lay across the above-mentioned two groups, indicating that they might have a similar aroma profile with both groups. This could be verified by the fact that discriminating compounds in PC1 were in a moderate concentration range between the above-mentioned two groups (Table S1 in Supplementary Materials).

### 2.3. Olfactory Characteristics of Each Strain in Small-Scale Fermentation

**Figure 2:**
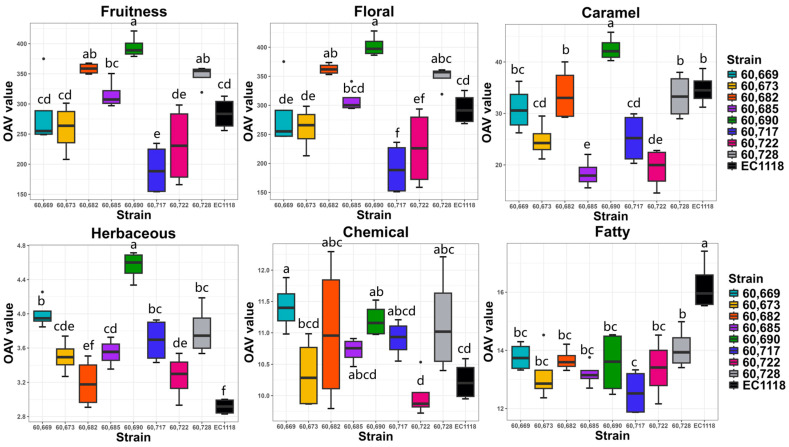
Aroma characteristics of indigenous and commercial yeasts based on the odor activity value (OAV) of aroma compounds in small-scale fermentations. (Indigenous strains: 60,669, 60,673, 60,717, 60,722, 60,728, 60,682, 60,685, 60,690; commercial strain: EC1118). Note: same lowercase letters indicates no significant difference (*p* < 0.05, Tukey’s post hoc test).

To better describe the sensory attributes of each yeast strain, the odor activity value (OAV) of each aroma compound was calculated based on its concentration and reported threshold [31]. Six predominant types of sensory attributes, namely, fruity, floral, caramel, herbaceous, chemical, and fatty, were calculated based on the sum of the OAV of each compound contributing to the same sensory attribute (Table S1 in Supplementary Materials). The different olfactory attributes were observed for each yeast strain (Figure 2). In terms of fruity note, strains 60,690, 60,682, and 60,728 had significantly higher OAV than the commercial yeast EC1118, whereas strain 60,717 had significantly lower OAV (*p* < 0.05) (Figure 2). This result was similar to the floral note and might be because these fruity aroma compounds were also characterized with a floral attribute (Table S1 in Supplementary Materials). The caramel attribute, however, was significantly higher only in strain 60,690, but significantly lower in strains 60,673, 60,685, 60,717, and 60,722 than in the commercial yeast EC1118 (*p* < 0.05) (Figure 2). Regarding the herbaceous note, all selected indigenous yeasts had a higher OAV than the commercial yeast (except strain 60,682). The herbaceous note was not a typical grassy character derived from C6 compounds because the concentration of the only C6 compound detected, namely, hexanol, was below its threshold (Table S1 in Supplementary Materials) [31]. Rather, it could be a floristic attribute derived from geranylacetone, which showed a higher OVA (Table S1 in Supplementary Materials). The chemical note, mainly attributed to higher alcohol, was generally higher in the selected indigenous yeast strains than in the commercial yeast (Figure 2). On the contrary, the fatty note, mainly due to the fatty acid, was significantly less pronounced (*p* < 0.05) in the selected yeast strains (Figure 2). Finally, strain 60,682 was selected as a yeast for pilot white and red wine fermentation trials because it embraced a similar aroma profile as both the commercial yeast and the other indigenous yeast strains. Strain 60,685 was selected as the other indigenous yeast for white and red wine fermentation trials because of its balanced positive olfactory attributes (fruity, floral, and caramel) but lower negative other attributes (herbaceous, chemical, and fatty). In addition, strain 60,690 was also selected for white wine pilot fermentation because of its remarkable fruity and floral attributes, which met the requirements of the typical white wine style.

### 2.4. The Performance of Selected Yeast Strains in Pilot Trials

In the pilot experiment, commercial yeast strains VL1 and VL2 were used for the fermentation of two white varieties (Sémillon and Riesling) and F15 for red varieties (Syrah and Cabernet Sauvignon) as controls. A principal component method, FAMD, was used to interpret the dataset as it contained both quantitative data (concentration of aroma compounds) and qualitative variables (grape varieties and yeast strains) [32]. It proved to be suitable for this dataset and was able to explain 65.9% (42% for dimension 1 and 23.9% for dimension 2) and 66.6% (46.8% for dimension 1 and 19.8% for dimension 2) of the total variance of the dataset for white and red varieties, respectively.

**Figure 3:**
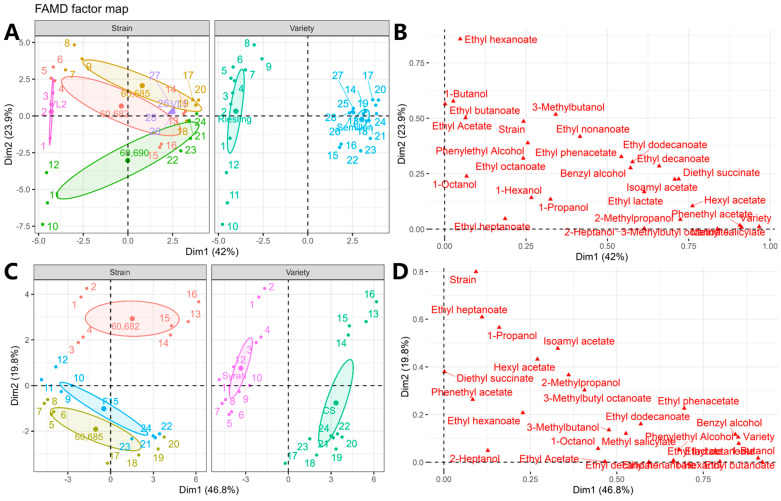
Factor analysis in mixed data (FAMD) showed the effects of strains and varieties (**A**,**C**) on the concentrations of aroma compounds in the pilot fermentations with indigenous and commercial yeasts, and the contributors of the model (**B**,**D**). (Indigenous strains: 60,669, 60,673, 60,717, 60,722, 60,728, 60,682, 60,685, 60,690; commercial strains: VL1, VL2, and F15).

Figure 3 clearly shows the effects of both varieties and yeast strains on the aroma profile in the pilot fermentation experiment. For both white and red wine, varietal effects were evident and demonstrated distinct separation on the first dimension (Figure 3A,C). For white wine, variety was the most important factor (>7% contribution, data not shown), dominating the distribution of the samples in the first dimension (Figure 3B), whereas for red wines, variety was the fourth most important factor (Figure 3D). This result was reasonable as Riesling is an aromatic variety whilst Sémillon is relatively neutral. For the quantitative variables, the most important aroma compounds for discriminating white varieties were methyl salicylate, phenethyl acetate, 3-methybutyl octanoate, and hexyl acetate (Figure 3B). They were all significantly higher in Sémillon wines (*p* < 0.05) than in Riesling wines (Table S2 in Supplementary Materials). For red varieties, ethyl butanoate, 1-butanol, ethyl octanoate, and benzyl alcohol were the most important discriminators (Figure 3D), and these compounds were significantly higher (*p* < 0.05) in Cabernet Sauvignon wines than in Syrah wines (Table S4 in Supplementary Materials).

Although a strong varietal effect was observed, yeast strains still exerted dominant effects by showing a marked separation of samples in the second dimension of the FAMD model for both white and red wines (Figure 3A,C). For the white wines, yeast strains showed a fifth rank in all discriminating variables (Figure 3B) with a contribution of more than 6% (data not shown). For red wines, yeast strains were the most prominent variables (Figure 3D), contributing more than 14% of the total variance (data not shown). Among the quantitative variables, phenylethanol was the most important aromatic compound (>11% contribution) for the separation of white wine samples in dimension 2, followed by 1-butanol, ethyl butanoate, 3-methylbutanol, and ethyl acetate (Figure 3B). Specifically, ethyl hexanoate and 1-butanol were significantly higher in white wines fermented with strains 60,682 and 60,685, and ethyl acetate was significantly higher in white wines fermented with strain 60,685 (*p* < 0.05) (Table S2 in Supplementary Materials). This phenomenon was consistent with the results obtained in small-scale fermentations, where 60,682 and 60,685 showed the highest or relatively higher production of the above compounds (Supplementary Table S1). In the red wines, ethyl heptanoate, 1-propanol, isoamyl acetate, hexyl acetate, and diethyl succinate were the most important aroma compounds affected by the yeast strains (>7% contribution). Among these compounds, only ethyl heptanoate, 1-propanol, and diethyl succinate showed a significantly higher concentration (*p* < 0.05) in red wines fermented with strain 60,682, while no significant difference was observed for isoamyl acetate or hexyl acetate (Table S3 in Supplementary Materials). However, of these compounds, only 1-propanol showed a similarly higher concentration in strain 60,682 as in the small-scale fermentation (Table S2 in Supplementary Materials).

To better understand the effect of variety and yeast strain and their interactions on the aroma compounds in the wines, two-way ANOVA was performed (Tables S2 and S3 in Supplementary Materials). In white wines, it was clear that the varietal effect was more influential than that of strain, as many more aroma compounds were highly significantly (*p* < 0.001) affected (Table S3 in Supplementary Materials). In addition, the interactive effect was also observed, but only for some aroma compounds (Table S3 in Supplementary Materials). By and large, Riesling showed much higher concentrations of fatty acid ethyl esters, whereas Sémillon showed much higher concentrations of higher alcohols and acetate esters (Table S3 in Supplementary Materials). With regard to yeast strain effects, the commercial yeast strain VL2 showed significantly higher concentrations of fatty acid ethyl esters compared to significantly higher concentrations of higher alcohols and acetate esters in indigenous yeasts (Table S3 in Supplementary Materials). For red wines, interactive effects between variety and strain were as significant as their main effects alone (Table S3 in Supplementary Materials). In general, more significantly higher concentrations of higher alcohols, fatty acid ethyl esters, and acetate esters were observed in Cabernet Sauvignon wine than in Syrah wine (Table S4 in Supplementary Materials). With regard to the performance of the yeast strains, the indigenous strain 60,682 showed a much more significantly higher concentration of higher alcohols and fatty acid ethyl esters than the other two strains (Table S3 in Supplementary Materials), which might indicate a distinctive aroma perception of the wine.

### 2.5. RATA Profiling of Pilot Experiment Wine

The sensory profile of the pilot experiment wine was evaluated using the RATA method, which has been shown to be a valid and rapid profiling method compared to the more costly and lengthy descriptive analysis (DA) [33]. A total of 23 attributes for white wine and 24 attributes for red wine were used for the RATA ballot (Table S4 in Supplementary Materials). According to the two-way ANOVA result, the varietal effect was still the main factor influencing the sensory profiles of the wines, especially for white wine, and a total of 14 attributes were screened with a significant difference (*p* < 0.05) by RATA (Table S5 in Supplementary Materials). These attributes were more pronounced in Riesling than in Sémillon, with the exception of “Apple/Pear”, “Citrus”, “Tropic Fruit”, and “Acidity” (Table S5 in Supplementary Materials). Regarding the effects of yeast strain, the notes of “Citrus” and “Stone Fruit” were significantly different (*p* < 0.05) according to RATA profiling, and they were higher in strain 60,685 than in the others (Table S5 in Supplementary Materials). The sensory result was in agreement with the chemical analysis, where more acetate esters were detected in strain 60,685 (Table S2 in Supplementary Materials). In contrast to the white wines, the main effects of both variety and yeast strain were limited for the red wines (Table S6 in Supplementary Materials). Only three sensory notes, “Green/Grassy”, “Tropic Fruit”, and “Finish”, were observed with significant differences (*p* < 0.05) due to the effects of variety, and wine acidity was significantly dominated by yeast strains (*p* < 0.05).

**Figure 4:**
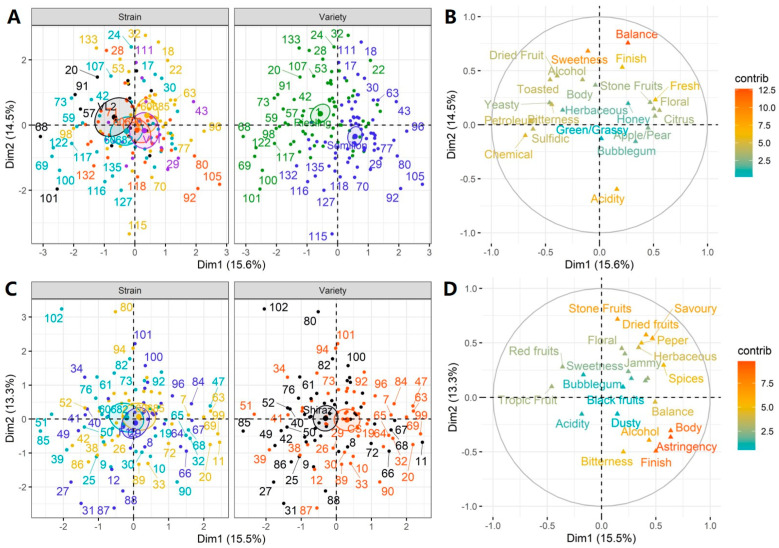
Multiple factor analysis (MFA) showing the effects of strains and varieties on wine sensory profiles with indigenous and commercial yeasts (**A**,**B**), and the corresponding sensory attributes (**C**,**D**) based on the rate-all-that-apply (RATA) method. (Indigenous strains: 60,669, 60,673, 60,717, 60,722, 60,728, 60,682, 60,685, 60,690; commercial strain: EC1118).

MFA was used to interpret these main effects of variety and yeast strain and, more importantly, to demonstrate the sensory profile comparable to commercial yeast. In Figure 4, varietal effects are clearly more pronounced, as the test samples could be almost completely (white variety in Figure 3A) or partially (red variety in Figure 3C) separated by variety, which was consistent with the two-way ANOVA results mentioned above. The main effects of yeast strain on wine sensory expression were relatively complex (Figure 4A,C), but some nuances could still be observed. For example, in the white variety, the commercial strain VL1 (in purple in Figure 4A) was located in the lower-right position in the coordinate system, whereas strain VL2 (in black in Figure 4A) was located in the upper-left position. This sensory profile was mapped in Figure 4B by showing more “Citrus”, “Apple/Pear”, “Stone Fruit”, and “Floral” olfactory notes and more “Fresh” and “Acidity” on the palate of strain VL1, compared to more “Dried Fruit”, “Toasted”, “Chemical”, and “Yeasty” sensations and more “Sweetness”, “Bitterness”, and “Body” in the mouthful perceptions of strain VL2. In terms of indigenous yeast, the distribution center of strain 60,682 (in cyan in Figure 4A) and 60,690 (in red in Figure 4A) was between the distribution center of strain VL1 and VL2, indicating that they had similar sensory profiles. However, samples of strain 60,685 (in yellow in Figure 4A) were distributed more to the right of the first dimension, indicating that they were also rated with “Honey”, “Citrus”, “Apple/Pear”, “Balance”, “Fresh”, and “Finish” sensations.

For red wines, the RATA profiles of strains 60,682 and 60,685 were similar to that of commercial yeast F15 (Figure 4C). However, subtle changes in their distribution indicated marginal difference in sensory characteristics. In Figure 4C, the center of strain 60,682 (in cyan in Figure 4C) was to the left of commercial strain F15, suggesting more notes of “Tropic Fruit”, “Red Fruit”, “Sweetness”, and “acidity” in such wines. In contrast, the center of strain 60,682 (in yellow Figure 4C) was to the right of commercial strain F15, indicating more “Spices”, “Pepper”, and “Floral” notes, and more “Body”, “Astringency”, and “Balance” being perceived in such wines. In general, however, the effect of yeast strain on the sensory profile of red wines was less pronounced than that of white wines, perhaps due to the more complex techniques used for red wine making, such as skin-contact maceration of red wines. Nevertheless, the indigenous yeasts still exerted noticeable effects on the sensory profile of both white and red wines in Huailai country and deserve further investigation.

## 3. Materials and Methods

### 3.1. Grape Sample

*Vitis vinifera* Sémillon (total sugar of 169.7 g/L, total acid of 6.9 g/L, pH 3.38), Riesling (total sugar 221.1 g/L, total equivalent tartaric acid of 6.6 g/L, pH 3.28), Cabernet Sauvignon (total sugar of 241.7 g/L, total equivalent tartaric acid of 4.5 g/L, pH 3.57), and Syrah (total sugar of 238.0 g/L, total equivalent tartaric acid of 5.7 g/L, pH 3.6) grapes (vintage 2020) were harvested in Shacheng region, Huailai country, Hebei province, China. The Sémillon and Riesling were harvested on 30 August and 9 September, respectively. The Cabernet Sauvignon and Syrah were harvested on 10 October and 21 September, respectively. The white varieties were pressed with a pneumatic press and then clarified with commercial pectinase (Lallemand, SA, Blagnac, France) before incubation with yeast. The red varieties were destemmed and selected manually before the commencement of yeast incubation and typical red wine fermentation.

### 3.2. Yeast Strain

The yeast strains were isolated during the spontaneous fermentation in vintage 2020 in COFCO Greatwall Chateau Sungod, Huailai country in China. Initially, a total of 120 indigenous yeast strains were isolated, and finally a total of eight strains (60,669, 60,673, 60,717, 60,722, 60,728, 60,682, 60,685, and 60,690) were selected for further experiments because they showed good tolerance to high alcohol and temperature, high concentrations of sugar and sulfur dioxide, and low pH (data not shown). All these strains were identified as *S. cerevisiae* by sequence analysis of the 5.8S-ITS rDNA (data not shown). All strains were stored at the Nutrition & Health Research Institute of COFCO Corporation in Beijing. A commercial yeast strain (*S. cerevisiae* EC1118, Lallemand, France) was used for comparison in a small-scale fermentation experiment. Two commercial yeast strains (*S. cerevisiae* VL1, VL2 and F15, Laffort, Floirac, France) were used as controls in pilot fermentation experiments for white wine making and red wine making, respectively.

### 3.3. Yeast Growing and Fermentation

The indigenous yeast strains were incubated in liquid yeast–peptone–dextrose (YPD) medium (10 g/L yeast extract, 20 g/L glucose, and 20 g/L peptone). The pasteurized (72 °C, 10 min) Sémillon was used for small-scale fermentation in sterilized 300 mL conical flasks with a fermentation lock. The incubation dosage was 3% *v*/*v* using the above yeast culture. Fermentation was controlled at a constant 25 °C. The fermentation process was monitored by measuring the weight (loss of CO_2_) every 12 h in a super-clean bench. A liquid quantity of 3 mL was also sampled and the OD value was measured. The small-scale fermentation was performed in tetraplicate.

Pilot scale fermentation was conducted using Sémillon, Riesling, Syrah, and Cabernet Sauvignon grapes. The fermentation conditions were typical winemaking techniques for white and red wines. For Sémillon and Riesling, the grapes were hand-selected and then pressed with pneumatic equipment; after pressing, the clarified grape juice was transferred into 100 L fermenters controlled at 14 °C for fermentation. For Syrah and Cabernet Sauvignon, the grapes were hand-selected, destemmed, crushed, and transferred into 100 L fermenters. Cold maceration was performed at 10 °C for 5 days, then the temperature was returned to 20 °C for yeast incubation. The cultivation of indigenous yeast was the same as previously described for small-scale fermentation. A 200 mL indigenous yeast YPD culture (10^6^ CFU/mL) was added to 5 L of corresponding grape juice and propagated for 24 h, then incubated into corresponding must. The incubation of commercial yeast was 0.2 g/L. The fermentation temperature for Syrah and Cabernet Sauvignon wine was 26–28 °C. Temperature and liquid density were measured continuously throughout the fermentation. Samples were collected after alcoholic fermentation (residual sugar < 4 g/L) and stored at −20 °C until analysis. Each fermentation was conducted in duplicate.

### 3.4. HS-SPME Condition and GC-MS Analysis

Headspace solid-phase microextraction (HS-SPME) was performed prior to GC-MS analysis using a published method [34]. A 2 cm DVB/CAR/PDMS 50/30 μm SPME fiber (Supelco, Bellefonte, PA, USA) was used on a CTC CombiPAL autosampler (CTC Analytics, Zwingen, Switzerland). The SPME fiber was activated at 250 °C, then 10 μL 4-methyl-2-pentanol (internal standard) and 1 g NaCl were added to a 5 mL wine sample in a 20 mL vial sealed with a PTFE-silicon septum. The SPME fiber was equilibrated at 40 °C for 30 min and then inserted into the sample vial at 40 °C with a stirring speed of 500 rpm for another 30 min to extract volatiles. Finally, the SPME fiber was inserted into a GC injector for 8 min to thermally desorbed volatiles for subsequent GC-MS analysis.

An Agilent 6890 gas chromatograph tandem with 5975C mass spectrometry coupled with a HP-INNOWAX capillary column (60 m × 0.25 mm × 0.25 μm, J&W Scientific, Folsom, CA, USA) was used for analysis. The conditions [34] were as follows: carrier gas (helium) of 1 mL/min, injector temperature of 250 °C, and splitless mode (0.75 min) used for injection. The GC heating program was as follows: initial temperature of 50 °C, held for 1 min, then increased to 220 °C at a rate of 3 °C/min. The ion source temperature and the quadrupole temperature were 250 °C and 150 °C, respectively. The MSD transfer line heater was 250 °C. The electron ionization (EI) mode at 70 eV was used, and the detector was set to full scan mode (*m*/*z* 30–350). Identification of the aromatic compounds was performed according to the retention time (RT), retention index (RI), and mass spectra of commercially obtained reference standards, which were compared with the standard NIST11 MS database. The identification was achieved automatically in the MassSpectral Deconvolution and Identification System (AMDIS) in combination with the NIST11 MS database. The quantification of each compound was performed using the calibration curve of corresponding standards prepared in model wine solution (2.0 g/L glucose, 7.0 g/L tartaric acid, 12% *v*/*v* ethanol, pH adjusted to 3.3 with 5 M NaOH).

### 3.5. HPLC Analysis

Glucose, fructose, glycerol, ethanol, and organic acids (acetic acid, citric acid, succinic acid, tartaric acid, and malic acid) were analyzed on an Agilent 1200 HPLC system (Agilent Technologies, Palo Alto, CA, USA) using a published method [35]; an Aminex ion exchange column (HPX-87H, 300 mm × 7.8 mm, Bio-Rad Laboratories, Hercules, CA, USA) was used, the mobile phase was 5 mM H_2_SO_4_ for isocratic elution, and the flow rate was 0.6 mL/min. For the analysis of glucose, fructose, glycerol, and ethanol, a differential refractive index detector (RID) was used, and the column oven was kept at 45 °C and the stop time was at 30 min; for the analysis of organic acids, a variable wavelength detector (VWD) was used (signal at 214 nm), and the column oven was kept at 60 °C and the stop time was at 30 min. The quantification of each compound was achieved using the calibration curve of each compound (prepared in distilled water). All samples were filtered through 0.22 μm polyethersulfone filters (Jinteng Experimental Equipment Co., Ltd., Tianjin, China) before HPLC analysis.

### 3.6. Analysis of Basic Enology Parameters of Wines

The basic enological parameters (residual sugar, alcohol content, titratable acid, pH, volatile acids, and sulfur dioxide) of wine were measured according to the national standards of the People’s Republic of China (GB/T 15038-2006).

### 3.7. Sensory Analysis

All sensory evaluations were conducted according to the ethical guidelines of the internal Ethics Committee of Beijing University of Agriculture (BUAEC-20220701), and informed consent was obtained from all participants. The wines were first tasted by a panel of wine experts, and a list of attributes was developed for a rate-all-that-apply (RATA) ballot. These listed attributes were randomly broken down by sensory modalities in the formal RATA questionnaire. Experienced wine evaluators (8 males and 9 females) between the ages of 24 and 32 were recruited. These evaluators either had a wine-related qualification (e.g., qualified with the Wine & Spirit Education Trust award) or were an experienced practitioner in the wine industry). Wine samples (30 mL) were randomly served in an opaque ISO standard glass following a complete block design with 3-digital code labeling. The replicate wine samples from the same fermentation experiment were evenly mixed for consistency. For the RATA evaluation, the evaluators were instructed to rate the intensity of the attributes using a seven-point scale (from “extremely low” = 1 to “extremely high” = 7). Distilled water and tasteless bread were provided as palate cleansers, and a 10 min break was requested between consecutive sessions for the white and red wine rating.

### 3.8. Statistical Analysis

One-way analysis of variance (ANOVA) was performed in R software (version 4.2.2) using Tukey’s post-hoc test in the “agricolae” package. Principal component analysis (PCA) was conducted in MetaboAnalyst 5.0 (https://www.metaboanalyst.ca/) (Assessed on 27 June 2022). Factor analysis in mixed data (FAMD) and multiple factor analysis (MFA) were performed using “FactoMineR” and “factoextra” packages. The other graphics were prepared in R using the “ggplot2” package.

## 4. Conclusions

In conclusion, the systematic selection and application of autochthonous yeast in Huailai country for white and red winemaking was carried out. The combination of chemical analysis and sensory evaluation showed distinctive characteristics of autochthonous yeast in modulating wine aroma and sensory profile. Overall, indigenous yeast could manipulate the aroma, chemical, and sensory profile of both white and red wines, and they performed differently with different varieties. Specifically, strain 60,685 imparted white wine with a higher concentration of esters and more “Citrus” and “Stone Fruit” sensory notes. Strain 60,682 endowed red wine with a higher concentration of esters and higher alcohol and acidity. In general, using indigenous yeast strains in Huailai country could potentially be a promising tool for the production of premium wines with regional characteristics.

## Figures and Tables

**Table 1:** ANOVA results of residual sugar, organic acids, ethanol concentration (g/L), and CO_2_ losses (g) of different yeast strains in small-scale fermentation trials.

Strain	CO_2_ Loss	Ethanol	OD_600_	Glucose	Fructose	Tartaric Acid	Malic Acid	Citric Acid	Glycerol	Succinic Acid	Lactic Acid	Acetic Acid
60,669	19.52 ± 0.81 a	92.25 ± 3.97 a	10.38 ± 0.04 ab	0	0.51 ± 0.72 a	1.75 ± 0.17 a	2.18 ± 0.1 b	0.67 ± 0 b	6.94 ± 0.18 ab	1.54 ± 0.02 a	0.12 ± 0.01 cd	0.14 ± 0.01 scd
60,673	18.76 ± 0.28 a	93.49 ± 2.43 a	8.54 ± 0.73 cd	0	1.41 ± 0.73 a	1.76 ± 0.13 a	2.21 ± 0.05 b	0.59 ± 0.01 c	6.23 ± 0.11 cd	1.28 ± 0.06 c	0.22 ± 0.01 a	0.22 ± 0.01 ab
60,682	18.63 ± 1.39 a	89.95 ± 6.09 a	11.19 ± 0.03 a	0	1.1 ± 0.05 a	1.76 ± 0.32 a	2.48 ± 0.17 a	0.85 ± 0.06 a	7.38 ± 0.51 a	1.58 ± 0.12 a	0.11 ± 0.01 d	0.16 ± 0 c
60,685	19.09 ± 0.02 a	93.87 ± 1.72 a	8.36 ± 0.33 d	0	1.25 ± 0.57 a	1.82 ± 0.09 a	2.16 ± 0.07 b	0.58 ± 0.01 c	6.1 ± 0.13 d	1.19 ± 0.13 c	0.21 ± 0.01 a	0.24 ± 0.01 a
60,690	19.56 ± 0.08 a	94.91 ± 0.06 a	9.5 ± 0.49 abcd	0	0.74 ± 0.16 a	1.84 ± 0 a	2.13 ± 0.01 b	0.66 ± 0 b	6.84 ± 0.04 abc	1.58 ± 0.01 a	0.14 ± 0.01 b	0.15 ± 0 cd
60,717	19.13 ± 0.95 a	91.22 ± 4.87 a	10.18 ± 0.42 abc	0	0.89 ± 0.41 a	1.69 ± 0.24 a	2.15 ± 0.12 b	0.67 ± 0.01 b	6.73 ± 0.44 abcd	1.35 ± 0.11 bc	0.13 ± 0.02 bcd	0.15 ± 0.01 cd
60,722	19.16 ± 0.66 a	92.45 ± 3.32 a	8.95 ± 1.8b cd	0	1.27 ± 0.17 a	1.76 ± 0.12 a	2.1 ± 0.05 b	0.68 ± 0.02 b	6.56 ± 0.3 bcd	1.29 ± 0.04 c	0.14 ± 0.01 bc	0.21 ± 0.03 b
60,728	19.09 ± 0.83 a	91.89 ± 3.98 a	9.32 ± 0.71b cd	0	1.05 ± 0.25 a	1.7 ± 0.21 a	2.2 ± 0.1 b	0.67 ± 0.01 b	6.77 ± 0.28 abcd	1.49 ± 0.05 ab	0.12 ± 0.01 cd	0.14 ± 0 cd
EC1118	19.64 ± 0.02 a	93.73 ± 0.55 a	9.73 ± 0.09 abcd	0	0.78 ± 0.01 a	1.74 ± 0.04 a	2.18 ± 0.02 b	0.66 ± 0 b	6.73 ± 0.1 abcd	1.55 ± 0.02 a	0.1 ± 0.01 d	0.13 ± 0 d

Note: Values of the same compound marked with the same lowercase letters indicates no significant difference (*p* < 0.05, Tukey’s post hoc test).

## Data Availability

The data used to support the findings of this study can be made available by the corresponding author upon request.

## Supplementary Materials

The following supporting information can be downloaded at: https://www.mdpi.com/article/10.3390/molecules29174279/s1. Figure S1: OD600 value (A) and CO_2_ loss (B) of each yeast strain during small-scale fermentation; Table S1: The concentration of the identified aroma compounds and their olfactory attributes in small scale fermentation; Table S2: Two-way ANOVA results and the comparison of the concentration of the identified aroma compounds in pilot fermentation experiment of white varieties; Table S3: Two-way ANOVA results and the comparison of the concentration of the identified aroma compounds in pilot fermentation experiment of red varieties; Table S4: List of attributes and description for RATA ballot; Table S5: Two-way ANOVA results and the comparison of the sensory attributes in pilot fermentation experiment of white varieties by RATA; Table S6: Two-way ANOVA results and the comparison of the sensory attributes in pilot fermentation experiment of red varieties by RATA. References [33,36,37,38] are cited in the Supplementary Materials.
