# Supplementary material for: The Role of Indigenous Yeasts in Shaping the Chemical and Sensory Profiles of Wine: Effects of Different Strains and Varieties
Source: Molecules. 2024 Sep 9;29(17):4279. doi: 10.3390/molecules29174279 (PMC11396908; doi:10.3390/molecules29174279)
Supplement: Supplementary file 1 [file molecules-29-04279-s001.zip › molecules-3184828-supplementary.pdf]

SUPPLEMENTARY INFORMATION FOR

The Role of Indigenous Yeasts in Shaping the Chemical and Sensory Profiles of Wine: Effects of Different Strains and Varieties

Xin-Ke Zhang[1, 2], Pei-Tong Liu[3], Xiao-Wei Zheng[3], Ze-Fu Li[4], Jian-Ping Sun[4], Jia-Shuo Fan[3], Dong-Qing Ye[5], De-Mei Li[1, 2], Hai-Qi Wang[4], Qing-Quan Yu[4],*, Zi-Yuan Ding[3],*

[1] Food Science and Engineering College, Beijing University of Agriculture, Beijing, 102206, China

[2] "The Belt and Road" International Institute of Grape and Wine Industry Innovation, Beijing University of Agriculture, Beijing, 102206, China

[3] Nutrition & Health Research Institute, COFCO Corporation, Beijing 102209, China

[4] COFCO Greatwall Chateau Sungod (Huailai) Co. Ltd., Hebei, 075499, China

[5] Guangxi Key Laboratory of Fruits and Vegetables Storage-Processing Technology, Guangxi Academy of

Agricultural Sciences, Nanning 530007, Guangxi, China

* Correspondence: yuqq@cofco.com and dingziyuan@cofco.com

Table of contents

Page

Figure S1. OD600 value (A) and CO2 loss (B) of each yeast strain during small-scale fermentation. 2

Table S1: The concentration of the identified aroma compounds and their olfactory attributes in small scale fermentation. 3

Table S2: Two-way ANOVA results and the comparison of the concentration of the identified aroma compounds in pilot fermentation experiment of white varieties. 5

Table S3: Two-way ANOVA results and the comparison of the concentration of the identified aroma compounds in pilot fermentation experiment of red varieties. 6

Table S4: List of attributes and description for RATA ballot. 7

Table S5: Two-way ANOVA results and the comparison of the sensory attributes in pilot fermentation experiment of white varieties by RATA. 8

Table S6: Two-way ANOVA results and the comparison of the sensory attributes in pilot fermentation experiment of red varieties by RATA. 9

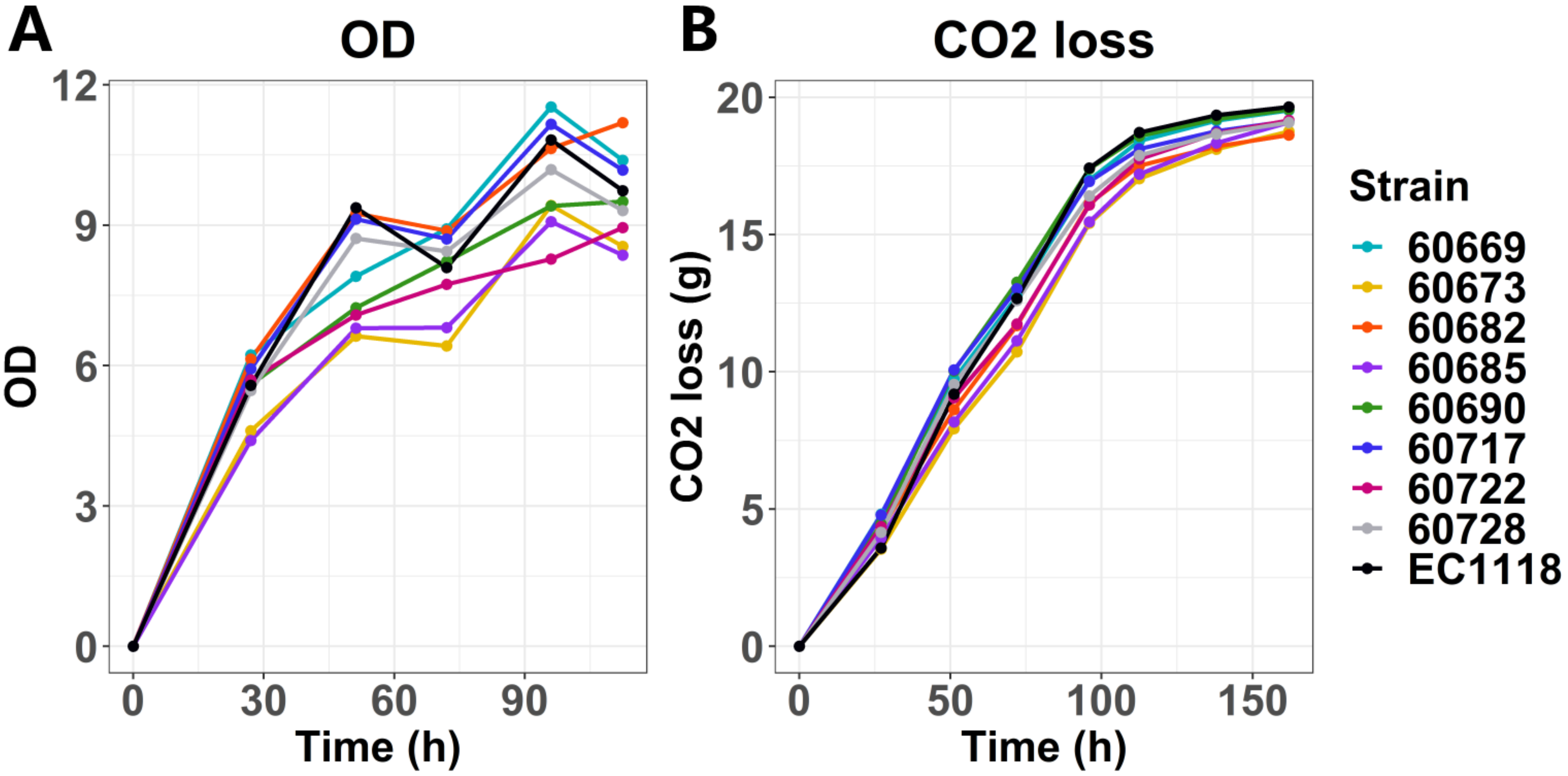

Figure S1. OD600 value (A) and CO_2 loss (B) of each yeast strain during small-scale fermentation.

Table S1: The concentration of the identified aroma compounds and their olfactory attributes in small scale fermentation.

Compound	Unit	Attributes	Threshold	60669	60673	60682	60685	60690	60717	60722	60728	EC1118
Propanol	mg/L	Fruity, Chemical	306[1]	21.1±0.8^{b}	19.1±1.5^{b}	**34.6±3.2^{a}**	20.4.6±0.3^{b}	21.4±0.79^{b}	20.96±0.51^{b}	18.79±1.21^{b}	21.0±2.1^{b}	**33.2±1.4^{a}**
Isobutanol	mg/L	Herbaceous, Chemical	75[1]	**43.25±1.2^{a}**	**47.5±3.8^{a}**	28.6±4.8^{b}	**47.3±1.7^{a}**	**41.4±0.8^{a}**	**49.7±7.9^{a}**	**42.7±8.2^{a}**	**42.9±4.4^{a}**	30.1±0.6^{b}
Butanol	µg/L	Chemical	150000[1]	1121.5±30.2^{bc}	608.9±30.9^{d}	**1744.4±302.6^{a}**	705.1±2.6^{d}	1314.1±24.9^{b}	916.6±227.6^{cd}	819.9±234.3^{cd}	1096.3±43.2^{bc}	1153.1±116.4^{bc}
Isopentanol	mg/L	Caramel, Chemical, Fatty	60[1]	259.7±11.2^{ab}	234.0±14.6^{bc}	246.7±27.1^{abc}	218.1±4.1^{c}	**267.4±7.7^{a}**	249.0±2.3^{abc}	225.3±6.1^{c}	257.4±19.6^{ab}	239.7±5.1^{abc}
Hexanol	µg/L	Herbaceous	1100[1]	144.9±10.4^{a}	149.8±16^{a}	138.6±15.1^{a}	146±5.6^{a}	138.3±12.1^{a}	135.9±10.5^{a}	142.5±14.2^{a}	135.4±14^{a}	127.2±2.7^{a}
1-Octanol	µg/L	Floral	800[1]	3.8±0.4^{a}	3±0.4^{a}	3.1±0.6^{a}	3±0.3^{a}	4±0.5^{a}	3.4±0.9^{a}	4.3±1.3^{a}	4.4±0.6^{a}	2.8±0.1^{a}
Benzyl alcohol	µg/L	Fruity, Caramel	200000[1]	46.9±51.4^{a}	45.7±10.2^{a}	70.8±13.6^{a}	22.2±5.4^{a}	52.6±9.7^{a}	20.1±5.2^{a}	45.3±17.4^{a}	30.3±20.2^{a}	26.4±18.9^{a}
Phenylethyl alcohol	mg/L	Floral	10[2]	48.4±1.7^{abc}	42.6±2.8^{bcd}	50.2±5.3^{ab}	41.0±2.4^{cd}	**53.0±1.3^{a}**	45.6±5.0^{abcd}	39.2±3.2^{d}	48.0.5±5.1^{abc}	48.3±2.9^{abc}
Ethyl acetate	mg/L	Fruity, Chemical	7.5[2]	44.4±1.3^{ab}	39.0±3.5^{bc}	**45.4±4.5^{a}**	43.7±0.9^{abc}	42.2±0.9^{abc}	40.8±0.8^{abc}	38.3±1.0^{c}	42.9±2.9^{abc}	40.2±2.2^{abc}
Isoamyl acetate	µg/L	Fruity	160[1]	**1897.3±92.9^{a}**	912.3±175.1^{e}	1495±191.1^{bc}	1116.6±22.5^{cde}	1975.4±92.7^{a}	1489.3±269.6^{bc}	1401.9±257.7^{bcd}	1788.3±125.9^{ab}	1050.7±82.4^{de}
Hexyl acetate	µg/L	Fruity, Floral	1500[1]	**5.6±0.2^{a}**	4.9±0.9^{abc}	3.9±0.7^{bc}	**6.1±0.3^{a}**	5.1±0.5^{ab}	4.1±0.5^{bc}	4±0.7^{bc}	**5.7±0.8^{a}**	3.6±0.3^{c}
Phenethyl acetate	µg/L	Fruity, Floral, Caramel	250[2]	645.7±30.2^{ab}	494.3±36.1^{bc}	539.5±46.1^{bc}	494.6±34.2^{bc}	**787.1±21.0^{a}**	514.8±123.7^{bc}	464.8±115.1^{c}	609.8±44^{bc}	489.7±18.7^{c}
Ethyl butanoate	µg/L	Fruity	400[1]	**186.8±5.6^{a}**	75.7±20.7^{e}	168.7±17.1^{abc}	112.1±8.9^{d}	154.7±6.9^{bc}	162.8±9.5^{abc}	158.6±3.8^{abc}	180.5±17.3^{ab}	148.3±17.0^{c}
Ethyl hexanoate	µg/L	Fruity	80[1]	288.3±6.5^{ab}	260.4±54.3^{b}	315±14.8^{ab}	**340.9±20.8^{a}**	285.5±10.9^{ab}	259.1±15.7^{b}	272.5±10^{b}	286±22.4^{ab}	295.9±32.9^{ab}
Ethyl lactate	µg/L	Fruity	154636[1]	190.2±29.6^{c}	**354.4±32.6^{a}**	164.3±25.8^{cd}	301.3±12.7^{b}	176.9±12.6^{cd}	196.1±12.4^{c}	134.6±6.5^{d}	203.3±34.0^{c}	177.3±10.4^{cd}
Ethyl octanoate	µg/L	Fruity, Floral	2[2]	503.4±116.5^{bcde}	471.4±77.6^{cde}	655.7±16.6^{ab}	580.9±46.8^{abcd}	**717.6±35.5^{a}**	331.4±79.8^{e}	413.3±127.6^{de}	629.3±33.9^{abc}	517.1±51.4^{bcd}
Ethyl nonanoate	µg/L	Fruity, Fatty		1.7±0.2^{b}	1.5±0^{b}	2.2±0.5^{ab}	1.5±0.1^{b}	**3.1±1.3^{a}**	1.5±0.1^{b}	1.6±0.2^{b}	2.1±0.8^{ab}	1.6±0.1^{b}
Ethyl decanoate	µg/L	Fruity	200[1]	891.3±503.7^{bc}	895.9±107.9^{bc}	1267.2±288.9^{ab}	664.6±83.3^{c}	1438±66.2^{a}	541.4±86.0^{c}	591.9±112.9^{c}	1223.5±116.7^{ab}	1027.5±88.9^{bc}
Ethyl dodecanoate	µg/L	Fruity, Fatty	1500[1]	84.6±8.9^{b}	170.4±13.5^{ab}	190.8±115.0^{a}	124.1±11.6^{ab}	96.5±4.3^{b}	78.8±1.3^{b}	82.4±1.8^{b}	91.6±6.9^{b}	90.9±2.6^{b}
Ethyl phenylacetate	µg/L	Fruity, Floral	650[3]	1.5±0.1^{b}	1.1±0.1^{c}	**2.2±0.2^{a}**	1.0±0.1^{c}	1.5±0.0^{b}	1.4±0.2^{b}	1.1±0.1^{c}	1.6±0.1^{b}	2.4±0.1^{a}
Methyl octanoate	µg/L	Fruity	200[1]	0.4±0.0^{ab}	0.4±0.0^{b}	**0.4±0.0^{a}**	**0.4±0.0^{a}**	**0.4±0.0^{a}**	0.4±0.0^{b}	0.4±0.0^{ab}	0.4±0.0^{ab}	0.4±0.0^{ab}
Isoamyl octanoate	µg/L	Fruity, Caramel, Fatty	125[1]	16.0±15.1^{cd}	**51.5±6.3^{a}**	10.5±1.2^{d}	38.1±4.5^{ab}	11.3±0.6^{d}	31.5±4.7^{bc}	18.3±12.9^{cd}	9.5±0.4^{d}	8.8±0.3^{d}
Methyl salicylate	µg/L	Fruity	40[4]	2.7±0.1^{ab}	2.7±0.0^{b}	2.7±0.0^{ab}	2.7±0.0^{b}	2.7±0.0^{b}	2.7±0.0^{b}	2.8±0.1^{a}	2.7±0.0^{b}	2.7±0.0^{b}
Hexanoic acid	mg /L	Fatty	0.42[1]	1.9±0.1^{bc}	1.8±0.1^{bc}	1.8±0.1^{bc}	2.0±0.5^{b}	1.6±0.1^{c}	1.7±0.7^{bc}	1.9±0.1^{bc}	1.8±0.1^{bc}	**2.3±0.2^{a}**
Octanoic acid	mg/L	Fatty	0.5[1]	2.4±0.1^{bc}	2.2±0.3^{bc}	2.6±0.1^{bc}	2.2±0.2^{bc}	2.5±0.3^{bc}	2.0±0.3^{c}	2.5±0.4^{bc}	2.6±0.0^{b}	**3.3±0.2^{a}**
Benzaldehyde	µg/L	Almond	2000[1]	3.1±1.0^{ab}	1.7±0.3^{b}	3.3±0.8^{ab}	2.8±1.7^{ab}	2.2±0.2^{b}	2.9±0.2^{ab}	2.5±0.3^{ab}	3.3±0.2^{ab}	**4±0.3^{a}**
Benzeneacetaldehyde	µg/L	Fruity	1[1]	23.9±4.2^{bc}	18.5±3.2^{cd}	27.5±4.7^{ab}	12.4±2.6^{d}	**34.9±2.3^{a}**	18.7±4.4^{cd}	13.6±3.6^{d}	26.6±4.0^{abc}	28.7±3.0^{ab}
Decanal	µg/L	Herbaceous	10[1]	1.8±0.2^{abc}	1.5±0.1^{abc}	1.2±0.9^{bcd}	**2.5±0.2^{a}**	1.5±0.8^{abc}	2.2±0.0^{ab}	2±0.1^{abc}	1±0.5^{cd}	0.3±0.0^{d}
trans Rose oxide	µg/L	Floral	0.2[1]	0.0±0.0^{a}	0.0±0.1^{a}	0.0±0.0^{a}	0.0±0.0^{a}	0.0±0.0^{a}	0.0±0.0^{a}	0.0±0.0^{a}	0.0±0.0^{a}	0.0±0.0^{a}

α-Terpineol	μg/L	Floral	250[1]	1.9±0.0[a]	1.8±0.0[a]	2±0.0[a]	1.8±0.1[a]	1.9±0.1[a]	2.7±1.8[a]	1.8±0.0[a]	1.8±0.1[a]	3.4±1.8[a]
Citronellol	μg/L	Floral, Caramel	100[1]	7.6±0.6[bcd]	6.8±0.3[d]	**8.8±0.1[a]**	7.1±0.3[cd]	**9.6±0.2[a]**	7.6±0.2[bcd]	8±0.5[b]	7.8±0.2[bc]	7.3±0.2[bcd]
trans Geranylacetone	μg/L	Fruity, Herbaceous	60[5]	153.8±6.1[ab]	118.2±8.5[bc]	127.4±11.0[bc]	117.2±8.3[bc]	**189.6±5.0[a]**	122.5±30.3[bc]	110.5±28.2[c]	146±10.8[bc]	117.2±4.6[bc]
cis Geranylacetone	μg/L	Fruity, Herbaceous	60[5]	2.8±2.0[bc]	3.5±0.4[abc]	**6.6±3.6[a]**	1.8±0.1[c]	5.7±0.4[ab]	3.7±2.3[abc]	1.7±0.1[c]	4.2±0.1[abc]	3.9±0.4[abc]

Note: Different letters denotes significant differences ($p < 0.05$), figures with underline indicated that they were above threshold; figures in bold indicated highest concentration compared to others (not necessarily significantly);

References used: [1] (Cai et al., 2014); [2] (Guth, 1997); [3] (Synos, Reynolds, & Bowen, 2015); [4] (Escudero, Gogorza, Melus, Ortin, Cacho, & Ferreira, 2004).

Table S2: Two-way ANOVA results and the comparison of the concentration of the identified aroma compounds in pilot fermentation experiment of white varieties.

Compounds (μg/L)	Unit	Threshold	Two-way ANOVA			Comparison between varieties		Comparison between yeast strains				
			Variety	Strain	Variety × Strain	Riesling	Sémillon	60682	60685	60690	VL1	VL2
1-Butanol	μg/L	150000[1]	***	***	**	376.98 ± 89.07a	350.11 ± 60.7a	**379.87 ± 39.67a**	**365.38 ± 92.29a**	285.34 ± 44.48b	**439.43 ± 34.47a**	**384.56 ± 35.66a**
1-Hexanol	mg/L	1.1[1]	***	***	***	2.16 ± 0.4b	**2.52 ± 0.3a**	2.55 ± 0.07a	2.49 ± 0.36a	2.30 ± 0.66a	2.00 ± 0.02a	2.3 ± 0.2a
1-Octanol	μg/L	800[1]	***	**	**	4.54 ± 2.12a	5.69 ± 2.41a	4.86 ± 0.95b	4.91 ± 1.99b	4.4 ± 2.33b	**9.18 ± 0.57a**	3.18 ± 0.57b
1-Propanol	mg/L	306[1]	*	***	***	20.75 ± 4.59b	**30.36 ± 10.66a**	23.59 ± 1.27b	**37.77 ± 12.51a**	19.04 ± 4.88b	27.94 ± 1.35b	20.09 ± 1.70b
2-Heptanol	μg/L	200[1]	*		.	0.39 ± 0.7b	**3.42 ± 1.85a**	1.68 ± 1.79a	2.81 ± 2.88a	2.69 ± 2.53a	1.13 ± 0.43a	1.53 ± 0.3a
2-Methylpropanol	mg/L	75[1]	***	***		21.88 ± 5.56b	**38.30 ± 5.75a**	**31.11 ± 6.59a**	**30.67 ± 1637.84a**	**31.25 ± 15.32a**	**42.29 ± 1.19a**	18.32 ± 17.50b
3-Methylbutanol	mg/L	60[1]	***	*		160.13 ± 424.46b	**191.69 ± 153.14a**	179.25 ± 119.52ab	**203.01 ± 5.92a**	161.82 ± 5.69ab	183.67 ± 7.85ab	148.47 ± 160.10b
3-Methylbutyl octanoate	μg/L	125[1]	***	***	**	3.27 ± 0.93b	**14.89 ± 3.15a**	8.03 ± 3.92b	9.31 ± 5.05b	9.96 ± 7.58b	**19.08 ± 0.91a**	3.38 ± 0.55b
Benzyl alcohol	μg/L	200000[1]	***			**191.67 ± 111.79a**	1.44 ± 3.15b	144.08 ± 180.22a	106.87 ± 132.3a	35.83 ± 42.18a	0.04 ± 0.09a	105.14 ± 16.12a
Diethyl succinate	mg/L	12000[1]		.		**12.18 ± 4.36a**	0.88 ± 0.25b	7.47 ± 8.51a	7.21 ± 7.89a	3.62 ± 3.21a	0.83 ± 0.25a	9.67 ± 1.72a
Ethyl acetate	mg/L	7.5[2]	**			56.68 ± 9.76a	59.75 ± 6.20a	57.52 ± 5.07ab	**60.97 ± 3.84a**	49.93 ± 9.34b	**65.01 ± 1.54a**	**65.74 ± 3.48a**
Ethyl butanoate	μg/L	400[1]	***	**	*	412.72 ± 115.07a	400.77 ± 75.35a	390.57 ± 48.83bc	467.31 ± 65.51ab	320.68 ± 105.63c	393.68 ± 15.09bc	**513.43 ± 70.58a**
Ethyl decanoate	mg/L	0.2[1]	***	*	**	**4.36 ± 1.23a**	1.59 ± 0.36b	2.79 ± 2.13b	2.98 ± 1.86b	2.07 ± 0.49b	1.99 ± 0.07b	**4.97 ± 0.81a**
Ethyl dodecanoate	mg/L	1.5[1]	***	**	.	**1.35 ±0.40a**	0.54 ± 0.16b	1.02 ± 0.70ab	1.04 ± 0.52ab	0.74 ± 0.11ab	0.40± 0.08b	**1.24 ± 0.14a**
Ethyl heptanoate	μg/L		***	**		**0.93 ± 0.32a**	0.55 ± 0.49b	**1.05 ± 0.39a**	0.58 ± 0.43b	0.4 ± 0.28b	0.43 ± 0.06b	**1.32 ± 0.27a**
Ethyl hexanoate	mg/L	0.08[1]		**		1.18 ± 0.38a	1.24 ± 0.18a	**1.32 ± 0.17a**	**1.43 ± 0.18a**	0.93 ± 0.35b	1.23 ± 0.01ab	1.15 ± 0.18ab
Ethyl lactate	μg/L	154636[1]	***	*	**	**844.78 ± 491.65a**	148.7 ± 28.74b	259.28 ± 108.62b	251.82 ± 174b	687.28 ± 706.01ab	173.83 ± 5.53b	**1144.21 ± 195.21a**
Ethyl nonanoate	μg/L		***	***	***	**3.65 ± 0.83a**	2.35 ± 0.58b	2.87 ± 1.25a	3.1 ± 1.3a	2.32 ± 0.27a	3.29 ± 0.19a	3.42 ± 0.25a
Ethyl octanoate	mg/L	0.002[2]	***	***		**3.11 ± 1.23a**	2.14 ± 0.24b	2.90 ± 1.15ab	2.59 ± 1.20ab	2.04 ± 0.39b	2.12 ± 0.03b	**3.45 ± 0.60a**
Ethyl phenylacetate	μg/L	650[3]	***	*		**0.38 ± 0.15a**	0.08 ± 0.06b	0.21 ± 0.24a	0.33 ± 0.21a	0.12 ± 0.09a	0.09 ± 0.01a	0.27 ± 0.04a
Hexyl acetate	mg/L	1.5[1]	***	***	***	0.58 ± 0.15b	**0.98 ± 0.12a**	0.88 ± 0.23a	0.84 ± 0.19a	0.71 ± 0.36a	0.92 ± 0.01a	0.64 ± 0.10a
Isoamyl acetate	mg/L	0.16[1]	***	***	***	4.17 ± 1.40b	**6.86 ± 0.93a**	5.56 ± 1.29ab	**6.58 ± 0.95a**	4.93 ± 2.68ab	**7.04 ± 0.14a**	4.07 ± 0.58b
Methyl salicylate	μg/L	40[4]	***			2.77 ± 0.2b	**3.45 ± 0.11a**	3.1 ± 0.4ab	**3.3 ± 0.31a**	3.15 ± 0.47ab	**3.35 ± 0.09a**	2.73 ± 0.15b
Phenethyl acetate	μg/L	250[2]	*	***	***	193.55 ± 55.02b	**424.38 ± 52.9a**	**338.95 ± 136.15a**	**371.88 ± 94.73a**	317.23 ± 169.6ab	**358.11 ± 9.85a**	161.26 ± 18.71b
Phenylethyl alcohol	mg/L	10[2]	***	*		16.96 ± 4.94b	**20.23 ± 3.12a**	19.17 ± 1.64b	**24.03 ± 1.24a**	1.64 ± 4.84bc	16.96 ± 1.52bc	14.09 ± 1.02c

Note: Different letters denotes significant differences ($p < 0.05$); figures in bold indicated significant higher concentration than others; '***', '**', '*', '.' and blank indicated the significant levels of < 0.001, < 0.01, < 0.05, < 0.1 and > 0.1, respectively.

References used: [1] (Cai et al., 2014); [2] (Guth, 1997); [3] (Synos, Reynolds, & Bowen, 2015); [4] (Escudero, Gogorza, Melus, Ortin, Cacho, & Ferreira, 2004).

Table S3: Two-way ANOVA results and the comparison of the concentration of the identified aroma compounds in pilot fermentation experiment of red varieties.

Compounds (μg/L)	Unit	Threshold	Two-way ANOVA			Comparison between varieties		Comparison between yeast strains		
			Variety	Strain	Variety × Strain	CS	Syrah	60682	60685	F15
1-Butanol	mg/L	150[1]	**	***	***	**1.70 ± 0.29a**	1.04 ± 0.13b	**1.58 ± 0.43a**	1.15 ± 0.25b	1.37 ± 0.41ab
1-Hexanol	mg/L	1.1[1]	***	***	***	**5.21 ± 0.58a**	3.72 ± 0.47b	4.84 ± 1.09a	4.26 ± 1.09a	4.28 ± 0.41a
1-Octanol	μg/L	800[1]	***	***	***	**15.74 ± 2.45a**	13.59 ± 1.4b	**15.37 ± 2.59a**	12.71 ± 1.19b	**15.91 ± 1.29a**
1-Propanol	mg/L	306[1]	***	***	***	52.42 ± 14.30a	48.31 ± 22.12a	**74.06 ± 5.58a**	37.27 ± 3.47b	39.77 ± 9.77b
2-Heptanol	μg/L	200[1]	***	***	.	3.66 ± 0.71b	**4.32 ± 0.53a**	4.1 ± 0.92a	4.15 ± 0.59a	3.7 ± 0.52a
2-Methylpropanol	mg/L	75[1]	***	*	***	66.42 ± 19.39b	**83.90 ± 20.47a**	48.94 ± 8.52b	**86.66 ± 11.00a**	**89.89 ± 11.50a**
3-Methylbutanol	mg/L	60[1]	***	***	***	**320.56 ± 27.70a**	278.97 ± 18.46b	287.33 ± 27.31a	29.57 ± 33.72a	31.68 ± 28.44a
3-Methylbutyl octanoate	μg/L	125[1]	***	***	***	**3.38 ± 1.19a**	2.55 ± 0.71b	**4.06 ± 0.83a**	2.9 ± 0.51b	1.94 ± 0.31c
Benzyl alcohol	μg/L	10000[1]	***	***	***	**524.9 ± 66.78a**	88.13 ± 6.83b	292.3 ± 221.21a	296.21 ± 228.17a	331.03 ± 261.84a
Diethyl succinate	μg/L	1200000[1]	***	***	***	13.92 ± 19.62a	24.94 ± 35.78a	**34.54 ± 32.35a**	0 ± 0b	23.74 ± 30.99ab
Ethyl acetate	mg/L	7.5[2]		***	*	**79.09 ± 8.03a**	70.97 ± 4.16b	75.92 ± 5.32a	74.49 ± 8.41a	74.67 ± 9.20a
Ethyl butanoate	μg/L	400[1]		**		**666.11 ± 108.77a**	375.77 ± 60.86b	592.64 ± 176.7a	474.82 ± 127.84a	495.35 ± 200.12a
Ethyl decanoate	mg/L	0.2[1]	***	***	***	**1.63 ± 0.50a**	0.79 ± 0.33b	1.38 ± 0.56a	1.29 ± 0.86a	0.96 ± 0.13a
Ethyl dodecanoate	μg/L	1.5[1]	***	***	***	**362.71 ± 96.64a**	222.96 ± 96.39b	**369.77 ± 152.86a**	242.64 ± 108.46b	266.09 ± 20.65ab
Ethyl heptanoate	μg/L		***	***	***	4.3 ± 0.81b	**5.88 ± 1.32a**	**6.17 ± 1.48a**	4.51 ± 0.9b	4.59 ± 0.97b
Ethyl hexanoate	mg/L	0.08[1]		***	**	1.28 ± 0.24a	1.19 ± 0.20a	**1.42 ± 0.24a**	1.13 ± 0.15b	1.16 ± 0.14b
Ethyl lactate	mg/L	154.64[1]	***	***	***	0.47 ± 0.16b	**1.05 ± 0.29a**	0.67 ± 0.33a	0.91 ± 0.52a	0.71 ± 0.21a
Ethyl nonanoate	μg/L		***	***	***	1.75 ± 0.45b	**21.88 ± 11.26a**	5.87 ± 6.11a	11.16 ± 10.6a	18.42 ± 17.5a
Ethyl octanoate	mg/L	0.002[2]	**		*	**1.82 ± 0.33a**	1.22 ± 0.26b	**1.82 ± 0.38a**	1.40 ± 0.44b	1.34 ± 0.29b
Ethyl phenylacetate	μg/L	650[3]	***	***	***	0.75 ± 0.24b	**5.8 ± 1.15a**	3.79 ± 3.16a	2.71 ± 2.4a	3.34 ± 2.75a
Hexyl acetate	μg/L	1500[1]	***	*	*	**76.17 ± 26.56a**	40.04 ± 12.63b	46.74 ± 8.78a	74.78 ± 35.28a	52.79 ± 25.66a
Isoamyl acetate	mg/L	0.16[1]	***	*	**	**2.85 ± 0.70a**	1.79 ± 0.32b	1.91 ± 0.24a	2.59 ± 0.63a	2.46 ± 1.08a
Methyl salicylate	μg/L	40[4]	***	*	*	3.93 ± 0.26b	**4.8 ± 0.39a**	4.37 ± 0.59a	4.39 ± 0.54a	4.32 ± 0.59a
Phenethyl acetate	μg/L	250[2]	***	***	**	58.06 ± 15.71a	61.83 ± 11.29a	52.34 ± 15.02b	**73.08 ± 2.3a**	54.41 ± 8.41b
Phenylethyl alcohol	mg/L	10[2]	***	***	***	**38.30 ± 3.98a**	32.52 ± 2.94b	**37.44 ± 5.04a**	32.70 ± 3.75b	36.10 ± 3.74ab

Note: Different letters denotes significant differences ($p < 0.05$); figures in bold indicated significant higher concentration than others; '***', '**', '*', '.' and blank indicated the significant levels of < 0.001, < 0.01, < 0.05, < 0.1 and > 0.1, respectively.

References used: [1] (Cai et al., 2014); [2] (Guth, 1997); [3] (Synos, Reynolds, & Bowen, 2015); [4] (Escudero, Gogorza, Melus, Ortin, Cacho, & Ferreira, 2004).

Table S4: List of attributes and description for RATA ballot.

Attributes	Description of attributes for white variety	Attributes	Description of attributes for red variety
Apple/Pear	Fresh cut of apples and pears	Green/Grassy	Freshly mowed/cut grass, green pepper/capsicum, or any combination of these
Bubblegum	Sweet aroma like candies/bubble gum	Red fruits	Raspberry, strawberry, red cherry, red currant, or any combination of these
Chemical	Home-using cleaning/disinfectant products or medical drugs	Jammy	Fresh jam
Toasted	Fresh toasted bread or grilled/hot woody aroma	Dusty	Earthy or dusty
Citrus	Lemon, orange, lime, grapefruit and any other citrus fruits, or any combination of these	Bubblegum	Sweet aroma like candies/bubble gum
Dried Fruit	Prune, date, sultana and any other dried fruits, or any combination of these	Floral	Rose, jasmine, honeysuckle, and any other perfumed flowers, or any combination of these
Floral	Rose, jasmine, honeysuckle, and any other perfumed flowers, or any combination of these	Herbaceous	Basil, thyme, oregano, mint, and any other fresh garden herbs, or any combination of these
Green/Grassy	Freshly mowed/cut grass, green pepper/capsicum or any combination of these	Spices	Anise, clove, cinnamon, liquorice, nutmeg, or any combination of these
Yeasty	Bread, dry yeast liquid or biscuit.	Stone Fruits	Peach and nectarine, or any combination of these
Honey	Honey	Chemical	Home-using cleaning/disinfectant products or medical drugs
Petroleum	Kerosene, gas station, petrol pump, or any combination of these	Black fruits	Blackberry, blackcurrant, plum, dark cherry, or any combination of these
Herbaceous	Basil, thyme, oregano, mint, and any other fresh garden herbs, or any combination of these	Dried fruits	Prune, raisins, fig and dried apricot, or any combination of these
Stone Fruits	Peach and nectarine, or any combination of these	Savoury	Preserved meats like pastrami, salami, sausage or any combination of these
Sulfidic	Onion, rotted egg, cooked cabbage, match, or any combination of these	Peper	White peper or black peper
Tropic Fruit	Pineapple, passionfruit, mango, or any combination of these	Tropic Fruit	Pineapple, passionfruit, mango, or any combination of these
		Oak/Toasted/Smokey	Aroma of oak or toasted oak
Body	The weight or thickness of the wine in the mouth	Body	The weight or thickness of the wine in the mouth
Sweetness	Sensation of sweet	Sweetness	Presence of sugar
Acidity	Sensation of sour	Acidity	Sensation of sweet
Alcohol	Hot or burnt perception caused by alcohol	Alcohol	Hot perception caused by alcohol
Bitterness	Bitterness	Bitterness	Bitterness
Fresh	Feeling of freshness, pleasure, energetic and refreshment	Astringency	Sensation of shrinks or constricts of tongue and oral cavity
Balance	Harmonious and balanced of each mouthful attributes	Balance	Harmonious and balanced of each mouthful attributes
Finish	The time which the flavor/feeling of the wine persists in the mouth	Finish	The time which the flavor/feeling of the wine persists in the mouth

Table S5: Two-way ANOVA results and the comparison of the sensory attributes in pilot fermentation experiment of white varieties by RATA.

Attributes	Two-way ANOVA			Comparison between varieties		Comparison between yeast strains				
	Variety	Strain	Variety × Strain	Riesling	Sémillon	60682	60685	60690	VL1	VL2
Apple/Pear	**	.		4.69±1.6b	**5.34±1.1a**	4.65±1.61a	5±1.37a	5.47±1.11a	4.94±0.97a	4.94±1.78a
Bubblegum				2.41±1.42a	2.75±1.12a	2.47±1.31a	2.85±1.31a	2.74±1.36a	2.35±1.06a	2.18±1.24a
Chemical	**			**3.59±1.28a**	2.94±1.08b	3.44±1.28a	2.88±1.23a	3.21±1.15a	3.29±1.21a	3.76±1.15a
Toasted	***			**2.41±1.16a**	1.57±0.72b	2.12±1.04a	1.79±0.84a	2±1.13a	1.59±0.71a	2.53±1.37a
Citrus	**	*		4.01±1.35b	**4.59±0.95a**	4.09±1.24ab	**4.74±1.21a**	4.47±1.02ab	4.24±0.97ab	3.59±1.33b
Dried Fruit	***			**2.91±1a**	1.99±0.82b	2.47±1.02a	2.35±1.1a	2.29±1.03a	2.41±0.87a	2.94±0.97a
Floral	.		.	3.18±1.09a	3.49±1.09a	3.09±1.24a	3.5±1.08a	3.32±1.12a	3.47±0.94a	3.35±0.93a
Green/Grassy				2.28±1.05a	2.5±1a	2.41±1.08a	2.32±1.01a	2.38±1.04a	2.35±1.17a	2.53±0.87a
Yeasty	***			**3.22±1.12a**	2.54±1.15b	2.97±1.22a	2.76±1.07a	2.79±1.37a	3.06±1.14a	2.94±1.03a
Honey				2.25±1.2a	2.41±0.98a	2.24±1.16a	2.41±1.02a	2.38±1.18a	2.29±0.99a	2.29±1.16a
Petroleum	***			**1.75±1.07a**	1.16±0.7b	1.59±1.08a	1.32±0.84a	1.38±0.85a	1.24±0.66a	1.82±1.24a
Herbaceous	**			**2.26±1.03a**	1.79±0.82b	1.88±0.88a	2.03±1.06a	2.15±0.93a	1.88±0.93a	2.24±1.03a
Stone Fruit	.	*		2.74±1.03a	3.06±1.03a	2.47±0.96b	**3.29±0.97a**	2.94±1.15ab	3.12±0.99ab	2.65±0.86ab
Sulfidic	***			**2.4±1.29a**	1.71±0.96b	2.15±1.31a	1.88±1.04a	2.09±1.14a	1.82±1.13a	2.35±1.41a
Tropic Fruit	***			3.29±1.35b	**4.19±1.54a**	3.65±1.67a	4.03±1.57a	3.47±1.31a	4.41±1.46a	3.24±1.3a
Body	.			3.94±1.1a	3.6±1.15a	3.68±1.25a	3.71±1.22a	3.79±1.01a	3.71±0.92a	4.12±1.22a
Sweetness	*			**1.69±0.78a**	1.41±0.6b	1.47±0.61a	1.71±0.84a	1.47±0.66a	1.47±0.62a	1.65±0.79a
Acidity	***			5.96±0.9b	**6.46±0.7a**	6.41±0.78a	6.09±0.87a	6.12±0.88a	6.41±0.71a	6±0.94a
Alcohol	***			**3.84±1.05a**	3.18±0.98b	3.38±1.07a	3.65±1.15a	3.35±0.95a	3.29±1.05a	4±1a
Bitterness	***			**3.04±1.11a**	2.29±0.79b	2.79±1.09a	2.53±0.93a	2.62±1.1a	2.35±0.86a	3.12±1.05a
Fresh	.			3.76±0.99a	4.1±1.08a	3.62±1.23a	4.15±1.05a	4±1.02a	4.18±0.81a	3.76±0.83a
Balance				3.41±0.83a	3.31±0.9a	3.24±0.89a	3.65±0.95a	3.29±0.76a	3.41±0.94a	3.12±0.7a
Finish				3.51±1.04a	3.41±1.01a	3.65±1.15a	3.71±0.97a	3.12±0.88a	3.29±0.92a	3.47±1.12a

Note: Different letters denotes significant differences ($p < 0.05$); figures in bold indicated significant higher concentration than others; '***', '**', '*', '.' and blank indicated the significant levels of < 0.001, < 0.01, < 0.05, < 0.1 and > 0.1, respectively.

Table S6: Two-way ANOVA results and the comparison of the sensory attributes in pilot fermentation experiment of red varieties by RATA.

Attributes	Two-way ANOVA			Comparison between varieties		Comparison between yeast strains		
	Variety	Strain	Variety × Strain	CS	Syrah	60682	60685	F15
Green/Grassy	***		*	**4.61±1.2a**	3.57±1.24b	4.18±1.29a	4.24±1.33a	3.85±1.35a
Red fruits				4.53±0.86a	4.67±0.89a	4.68±0.94a	4.68±0.88a	4.44±0.79a
Jammy				3.69±0.99a	3.65±0.93a	3.59±0.86a	3.68±0.94a	3.74±1.08a
Dusty				1.53±0.86a	1.41±0.67a	1.41±0.61a	1.5±0.71a	1.5±0.96a
Bubblegum			**	1.96±0.77a	2.18±0.82a	2.03±0.83a	2.06±0.74a	2.12±0.84a
Floral				4.2±1.18a	4.16±1.08a	3.94±1.01a	4.47±1.05a	4.12±1.27a
Herbaceous				3.59±1.04a	3.37±1.22a	3.5±1.21a	3.65±1.01a	3.29±1.17a
Spices				3.14±0.94a	2.94±1.05a	3.12±1.12a	3.06±0.81a	2.94±1.04a
Stone Fruit				3.82±1.07a	3.71±1.1a	3.79±1.15a	3.88±1.01a	3.62±1.1a
Chemical				2.27±1.25a	2.1±0.67a	2.15±1.16a	2.29±1.06a	2.12±0.77a
Black fruits				5.63±1.18a	5.49±0.73a	5.41±0.96a	5.56±1.13a	5.71±0.84a
Dried fruits				3.73±0.98a	3.53±0.99a	3.47±0.75a	3.62±0.92a	3.79±1.23a
Savoury				3.57±1.17a	3.35±0.98a	3.5±1.08a	3.38±1.07a	3.5±1.11a
Peper				3.31±0.95a	2.98±1.05a	3.15±1.05a	3.15±0.96a	3.15±1.05a
Tropic Fruit	*			1.76±0.68b	**2.16±0.83a**	1.91±0.83a	1.97±0.8a	2±0.74a
Oak/Toasted/Smokey				2.02±0.97a	2.08±0.84a	2.21±0.91a	2.06±0.81a	1.88±0.98a
Body				4.8±0.83a	4.55±0.92a	4.53±0.96a	4.82±0.94a	4.68±0.73a
Sweetness				1.41±0.8a	1.39±0.7a	1.21±0.64a	1.59±0.89a	1.41±0.66a
Acidity		**		5.31±1.07a	5.51±1.05a	**5.79±0.91a**	5.44±1.21ab	5±0.89b
Bitterness				3.82±1.29a	3.59±0.92a	3.56±1.02a	3.53±1.21a	4.03±1.09a
Alcohol	.			4.1±1.12a	3.78±0.73a	3.76±1.05a	4.06±0.98a	4±0.82a
Astringency				4.25±1.09a	4.12±1.07a	4.21±1.2a	4.15±1.02a	4.21±1.04a
Balance			.	5.2±0.75a	5.08±0.8a	4.97±0.72a	5.26±0.71a	5.18±0.87a
Finish	*			**4.88±0.82a**	4.49±0.86b	4.5±0.86a	4.79±0.84a	4.76±0.85a

Note: Different letters denotes significant differences ($p < 0.05$); figures in bold indicated significant higher concentration than others; '***', '**', '*', '.' and blank indicated the significant levels of < 0.001, < 0.01, < 0.05, < 0.1 and > 0.1, respectively.

References:

Cai, J., Zhu, B.-Q., Wang, Y.-H., Lu, L., Lan, Y.-B., Reeves, M. J., & Duan, C.-Q. (2014). Influence of pre-fermentation cold maceration treatment on aroma compounds of Cabernet Sauvignon wines fermented in different industrial scale fermenters. *Food Chemistry*, 154, 217-229.

Escudero, A., Gogorza, B., Melus, M., Ortin, N., Cacho, J., & Ferreira, V. (2004). Characterization of the aroma of a wine from Maccabeo. Key role played by compounds with low odor activity values. Journal of Agricultural and Food Chemistry, 52(11), 3516-3524.

Guth, H. (1997). Quantitation and sensory studies of character impact odorants of different white wine varieties. Journal of agricultural and food chemistry, 45(8), 3027-3032.

Synos, K., Reynolds, A. G., & Bowen, A. J. (2015). Effect of yeast strain on aroma compounds in Cabernet franc icewines. *LWT - Food Science and Technology*, 64(1), 227-235.
